# Vitamin K2 Needs an RDI Separate from Vitamin K1

**DOI:** 10.3390/nu12061852

**Published:** 2020-06-21

**Authors:** Asim Cengiz Akbulut, Angelina Pavlic, Ploingarm Petsophonsakul, Maurice Halder, Katarzyna Maresz, Rafael Kramann, Leon Schurgers

**Affiliations:** 1 Department of Biochemistry, Cardiovascular Research Institute Maastricht, 6200MD Maastricht, The Netherlands; a.akbulut@maastrichtuniversity.nl (A.C.A.); a.pavlic@maastrichtuniversity.nl (A.P.); p.petsophonsakul@maastrichtuniversity.nl (P.P.); 2 Division of Nephrology, RWTH Aachen University, 52074 Aachen, Germany; mhalder@ukaachen.de (M.H.); rkramann@ukaachen.de (R.K.); 3 International Science & Health Foundation, 30-134 Krakow, Poland; katarzyna.maresz@nutricon.eu

**Keywords:** vitamin K, vitamin K1, vitamin K2, RDI, menaquinone, vitamin K-dependent proteins

## Abstract

Vitamin K and its essential role in coagulation (vitamin K [Koagulation]) have been well established and accepted the world over. Many countries have a Recommended Daily Intake (RDI) for vitamin K based on early research, and its necessary role in the activation of vitamin K-dependent coagulation proteins is known. In the past few decades, the role of vitamin K-dependent proteins in processes beyond coagulation has been discovered. Various isoforms of vitamin K have been identified, and vitamin K2 specifically has been highlighted for its long half-life and extrahepatic activity, whereas the dietary form vitamin K1 has a shorter half-life. In this review, we highlight the specific activity of vitamin K2 based upon proposed frameworks necessary for a bioactive substance to be recommended for an RDI. Vitamin K2 meets all these criteria and should be considered for a specific dietary recommendation intake.

## 1. Introduction

Vitamin K was first discovered almost a century ago, as was its essential role in coagulation [1]. Since then, vitamin K and vitamin K-dependent proteins have been illustrated to have a multitude of various functions beyond coagulation [2]. Vitamin K exists in various isoforms, namely, phylloquinone, commonly referred to as vitamin K1 (VK1), and menaquinones, also known as vitamin K2 (VK2). VK2 can be further classified into various subtypes, and the most well-known ones are menaquinone-4, -7, -8, and -9 (MK-4, MK-7, MK-8, MK-9) [3,4,5]. VK1 is found mainly in leafy green vegetables, such as spinach, Swiss chard, and kale, to name a few [6,7]. VK2 is primarily produced by bacteria, so it is found in high concentrations in fermented foods, such as pickled vegetables and cheeses. Relative amounts of VK2 can be found in particular meats of farmed animals. This is due to menadione supplementation in animal feed, given to prevent fractures, as well as from the naturally occurring conversion of VK1 to VK2 as MK-4 [8,9,10,11].

Vitamin K is essential for maintaining proper body function, and a deficiency has been linked to age-related diseases [12]. Furthermore, vitamin K has its own Recommended Daily Intake (RDI) based on the median intake of VK1 in adults in the US [13]. However, accumulating evidence points towards a role of VK2 that differs from VK1. This is in relation to the absorption, half-life profiles, carboxylation efficacy of VK2 on vitamin-K dependent proteins, and even non-carboxylated mediated processes that VK1 lacks [14,15]. Therefore, a clear differentiating mode of action of VK2 from VK1 has come to light [9,16]. The action of MK-7 as a cofactor in the carboxylation of its dependent factors is so strong that any supplementation should be avoided in patients prescribed vitamin K antagonists, whereas no such caution is needed for the ordinary dietary intake of VK1 [17]. Additionally, VK2 has been shown to play a role in improving outcomes for osteoporosis, atherosclerosis, cancer, and inflammatory diseases [2]. Accumulating data from both basic science and clinical studies demonstrate that the beneficial effects of VK2 are not covered by current RDI guidelines. The consequence of looking past these data has resulted in insufficient intakes. Furthermore, unlike specific fermented foods, such as natto, commonly consumed in certain regions of Japan, VK2 intake based on its presence in food can generally be considered low in the rest of the world [18,19]. Therefore, there is a need for including VK2 in recommendations in addition to VK1.

In 2014, a nine-criteria standard was formulated to assess whether there are sufficient grounds for a nutraceutical to be considered for an RDI [20]. The criteria encompass (1) an accepted definition; (2) a reliable analysis method; (3) a food database with known amounts of the bioactive; (4) cohort studies; (5) clinical trials on metabolic processes; (6) clinical trials for dose–response and efficacy; (7) safety data; (8) systematic reviews and/or meta-analyses; and lastly, (9) a plausible biological rationale. By evaluating current knowledge and studies, either performed or still ongoing, we assessed whether VK2 meets these nine criteria.

## 2. Generally Accepted Definition

**Figure 1:**
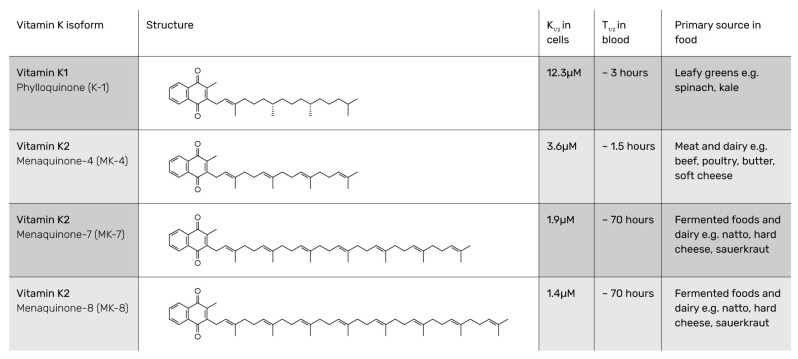
Name, structure, K1/2, T1/2, and main sources of vitamin K1 and major vitamin K2 isoforms [25].

VK2 is a group of compounds composed of a methylated naphthoquinone ring with an unsaturated sidechain and varying isoprenyl units (from 1 to 13, which defines n in the MK-n abbreviation). VK2 differs from VK1, as the latter only has one unsaturated sidechain unit (Figure 1). The structural difference between VK1 and VK2 has been known and appreciated in research since the beginning of the 20th century [21,22,23,24]. The different isoforms of VK2 have individual Chemical Abstracts Services (CAS) registry numbers: MK-4 863-61-6; MK-7 2124-57-4; MK-8 523-38-6; and MK-9 523-39-7.

## 3. Reliable Analysis Method Complies with Definition

An accepted standard for distinguishing VK1 and VK2 is established through the use of reversed-phase high-performance liquid chromatography (HPLC). This technique is used frequently to analyze vitamin K content in food and allows for the quantification of separate isoforms of VK2. A European standard method (EN 14148:2003) exists to determine VK1 by HPLC, but no official method has been registered for measuring VK2. However, various reports have used HPLC to accurately identify VK2 in meats, dairy, or fermented foods [8,26,27,28,29,30,31]. The development of the vitamin K external quality assurance scheme (KEQAS) for harmonization of serum VK1 measurements has improved the comparability of clinical and nutritional studies [32]. The variety of methods for detecting vitamin K and its analogs has been recently described in detail by others [33]. Recently, a combination of liquid chromatography with triple quadrupole mass spectrometry (LC-MS/MS) was reported to be able to quantify VK1 and VK2 isoforms MK-4 and MK-7 from human serum in different populations [34,35,36,37]. Whether this is the beginning of the establishment of a gold standard remains an open question, and further efforts are being made to include VK2 in the KEQAS with pilot studies on MK-4 and MK-7 ongoing (www.keqas.com).

## 4. Food Databases on Vitamin K2

VK2 can be found in diets worldwide. The majority of investigations have been into diets from the USA, Europe, and Japan, where the main sources are fermented foods, cheeses, and meats. The European Food Safety Authority (EFSA) and the United States Department of Agriculture (USDA) provide a dietary overview of vitamin K distribution in food based on studies in the aforementioned regions [38,39], in the US [28,40,41] (USDA, 2015), and in Japan [30,42]. Table 1 summarizes relevant differences in concentrations of VK1 and VK2 in food [2].

## 5. Prospective Cohort Studies

Almost 1000 years of northern Japanese cuisine has included ‘natto’, the world’s richest food source of VK2. A VK2-rich diet has existed for approximately 30 generations without any adverse side effects. This has been postulated as the reason that Japan has a lower fracture risk and stronger bone density than other countries. This also holds true within Japan, as areas of higher natto consumption have reduced bone loss than others [43,44,45]. Recent VK2 supplementation studies have given dosages as high as 135 mg per day with no adverse side effects reported [46]. In fact, Japanese medical authorities prescribe 45 mg/day of VK2 (15 mg MK-4 taken three times per day) supplementation to osteoporotic women [47]. Internationally, numerous individuals have participated in trials involving VK2 with no adverse side effects reported. This includes trials using doses ranging from 10 µg to 45 mg/day for several years. The most recent study in Japanese postmenopausal women showed that even doses of 350 µg MK-7 per week showed a reduced risk of osteoporotic fractures [17,46,48]. Aside from supplementation, vitamin K deficiency is well established in hemodialysis patients; this has implicated vitamin K as the link between vascular calcification, bone mineral density, and fracture rate [49,50]. With these combined, vitamin K2 supplementation suggests a potential beneficial trend that has been observed in reducing fracture risk, cardiovascular disease, as well as the development of type II diabetes and chronic kidney disease (CKD) [51,52,53,54,55,56,57,58,59,60,61,62,63].

## 6. Clinical Trials on Metabolic Processes

Vitamin K is a fat-soluble vitamin, which primarily acts as an unequivocal cofactor in the carboxylation of vitamin K-dependent proteins (VKDPs) [64]. After dietary intake, vitamin K, along with certain pancreatic hydrolysis products, is emulsified by bile salts as part of the digestion process. The absorption of vitamin K takes place in the small intestine, where it is taken up by the enterocytes and packaged into chylomicrons [65]. Lipoprotein lipase accounts for catabolizing these chylomicrons and facilitating further uptake [66]. Vitamin K, which remains in the lipophilic core after the catabolic process, enters the circulation and is transported to the liver by triglyceride-rich lipoproteins [67].

This is when the actions of VK1 and VK2 begin to vary. VK1 is preferentially retained in the liver and rapidly excreted, whereas VK2 acts within the liver and is transported into the circulation. As a result, VK2 is available to the whole body, including for reuse in the liver. This is due to the transportation processes carried out by low-density lipoproteins [68]. The absorption rate of VK2 in the small intestine is increased at a higher concentration of bile salt and unsaturated fatty acid [69]. It is known that vitamin K is better absorbed when consumed with fat [70]. VK2 has a better absorption profile in comparison to VK1, which showed a large inter-individual variation in plasma concentration after ingestion [8,71]. Further, the absorption profile of vitamin K varies between isoforms; in brief, only 10–15% of VK1 is absorbed by the body, whereas isoform MK-7 is more completely absorbed by the body [9].

MK-7 seems to have the most potent efficacy in terms of absorption and bioavailability [72]. It is absorbed within 4 h of ingestion and exhibits 10-fold higher postprandial serum concentration than VK1 [8]. MK-7 has a longer half-life (72 h) and lasts up to 144 h in the circulation, while VK1 is rapidly cleared from plasma [8]. VK1 absorption from green vegetables is less than 10 percent of the consumed amount, and the half-life is calculated to be 3 h [8,73,74]. Studies on the excretion of VK2 in humans are lacking. At present, only one such study is available, and it reports that VK2 isoform MK-4 is excreted by bile and is removed from the liver faster than VK1 [75]. However, it is important to note that VK1 can be converted into VK2 isoform MK-4 [76].

**Figure 2:**
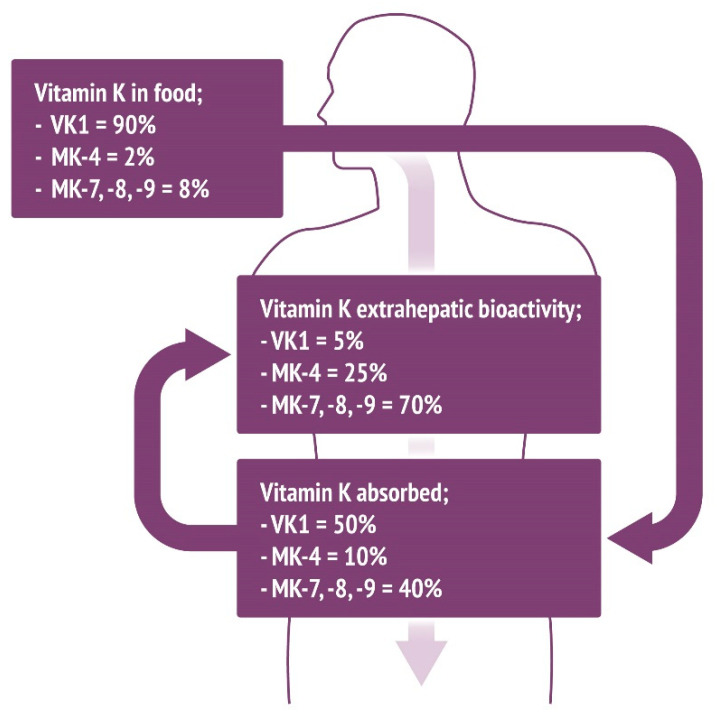
Intake of vitamin K and percentage of total absorbed vitamin K based upon estimated approximations of levels of vitamin K in the Western diet and previously determined absorption values. Given that VK1 is only 10–15% absorbed and that VK2 analogs are more completely absorbed, actual vitamin K levels vary significantly compared to the food content. Further to this, consumption of VK2 isoforms MK-7, -8, and -9 contributes to the majority of extrahepatic processes regulated by VKDPs.

Based on estimated dietary consumption, VK1 accounts for 90% of the total vitamin K in the diet [8]. However, given that only 10–15% of this is absorbed in the digestive tract [8], VK1 accounts for 50% of the total absorbed vitamin K. Based on absorption profiles, we hypothesize that MK-4 accounts for 10%, with MK-7, -8, and -9 making up 40% of total absorbed vitamin K. Based on the known aforementioned modes of vitamin K activity, MK-7, -8, and -9 account for 70% of total extrahepatic activity, with VK1 only contributing to 5% of this (Figure 2).

At a sub-cellular level, vitamin K epoxide reductase (VKOR) and gamma-glutamyl carboxylase (GGCX) are required to facilitate the redox cycle of vitamin K, which concomitantly carboxylates VKDPs to become active [64]. This process takes place in the endoplasmic reticulum and exerts its function at the cell surface or in the extracellular matrix of specific tissues [77].

Clinical trials have further revealed baseline deficiency of vitamin K in patients from CKD and hemodialysis cohorts [60,61,62,63]. Markers for vitamin K deficiency such as dp-ucMGP (dephospho-uncarboxylated atrix Gla protein), plasma phylloquinone, ucOC (undercarboxylated osteocalcin), and PIVKA-II (protein induced by vitamin K absence or antagonism factor II) have been found to be chronically higher in patient cohorts [58,59,60,61]. Both bone density and fracture rate in CKD patients have been correlated to vitamin K status [58,59]. Interestingly, one such study noted decreased phylloquinone plasma levels in patients who have experienced fractures [61]. Further mechanistical exploration of the in vitro role of vitamin K in mesenchymal stem cell differentiations might reveal insights into VK1 and VK2 in bone formation and fracture healing [78].

## 7. Clinical Trials on Efficacy and Dose–Response

In clinical trials, VK2 supplementation consistently has shown reductions in dp-ucMGP, PIVKA-II, and ucOC. Reduced levels of these proteins are acceptable biomarkers for countering vitamin K deficiency. Modulation of dp-ucMGP levels by daily supplementation has been demonstrated with 360 μg of MK-7 per day. This decreased dp-ucMGP levels by 86% with no adverse effects in the cohort [79]. Another similar trial showed a significant decrease in dp-ucMGP levels in hemodialysis patients with doses of MK-7 as high as 1080 μg administered three times per week [80]. No negative effects were observed, although dropouts did occur due to ‘the unpleasant smell of the tablets’ by which MK-7 was administered. PIVKA-II has been used notably in assessing vitamin K status in newborns. One such study found that 25 μg VK1 supplementation did not affect PIVKA-II status, whereas only 12 μg VK1 supplementation or placebo had a significant reduction in PIVKA-II status [81]. ucOC has also been reduced by VK1 and VK2 supplementation with no adverse effects in participants [82,83,84]. Interestingly, ucOC modulation by 45 mg per day of VK2 in an osteoporosis cohort study coincided with the prevention of fractures in the cohort [85]. Further, a landmark study demonstrated that MK-7 supplementation in healthy patients and hemodialysis patients could significantly decrease dp-ucMGP and ucOC levels while decreasing PIVKA-II status. A variety of doses were administered, and 360 μg/day was both the highest and most effective with no toxic effects present [52]. In Japan, natto is frequently eaten in doses of 50 g, which equals about 500 μg MK-7/day, with no reported adverse toxic or dose-dependent effects.

## 8. Safety Data

Concentrations of VK2 isoforms have been found to be as high as 1000 μg per 100 g natto [18]. This demonstrates the safety of VK2 in this range of daily intake. Furthermore, multiple studies have been performed using VK2 concentrations of up to 45 mg/day [45,85,86]. One isoform of VK2 has received a Generally Recognized as Safe (GRAS) status by the FDA. MenaQ7, a commercial form of MK-7, was judged by an independent panel to be allowed as an ingredient in food products [87].

Various toxicological studies on menaquinones have been performed. The European Food Safety Authority (EFSA) does not differentiate between VK2 isoforms and allows for this due to the similar metabolic conversion of all derivatives [88]. Animal studies revealed a lack of toxic effects after administration of one-time doses of MK-7 ranging up to 2000 mg/kg [89]. The confirmation of the safety of MK-7 can be derived from available data of clinical studies. In Japan, MK-4 in the dose of 45–90 mg/day has been used in the treatment of osteoporosis for many years without any reports of adverse effects [90].

## 9. Systematic Reviews and/or Meta-Analyses

Current available systematic reviews and meta-analyses on VK2 supplementation show a strong correlation supporting bone health [88]. Supplementation of VK2 reduces bone loss and decreases incidence of fractures among the Japanese population [52]. Further studies have confirmed this positive effect of VK2 on fracture risk. A large meta-analysis that included 19 randomized controlled trials from a heterogeneous population revealed a significant improvement of vertebral bone mineral density (BMD) in postmenopausal women with osteoporosis who were supplemented with either MK-4 at 45–90 mg/day or MK-7 at 180 μg/day [90].

In healthy postmenopausal women, three-year MK-7 supplementation of 180 μg/day greatly reduced age-related decline in BMD, increased bone strength, and reduced vascular stiffness [50,89,91]. According to the American Family Physician toolkit for Evidence-Based Medicine, VK2 is supported by level I and II evidence for osteoporosis [4]. Moreover, VK2 is supported by level II evidence in the prevention of coronary calcification and cardiovascular disease [4]. Despite numerous individual studies on the VK2 protective role in the vasculature, systematic reviews and meta-analyses have been unable to demonstrate a clinically significant link. Although not significant, there is a correlation of VK2 supplementation with improved cardiovascular health [92,93]. This might be due to study selection criteria, heterogeneity in the participant group, and specific interventions. The challenges in nutritional science research domains often include quantitative and qualitative nutrition assessment and variations in the source of dietary intake, study design, and study duration [94]. Moreover, different bioactive forms and bioavailability, which may be altered by the co-ingestion of other foods and supplements, should be highlighted as one of the issues in systematic reviews that are conducted to support the development of nutrient reference values [95].

## 10. Plausible Biological Rationale

The main role of VK2 is as a cofactor for the gamma-glutamyl carboxylation of hepatic and extrahepatic proteins [96,97,98,99]. As mentioned previously, this reduces dp-ucMGP, ucOC, and PIVKA-II levels, which are proteins that have a role in maintaining vascular, bone, and hepatic health. The bone–vascular axis has been recently described, and the role of extrahepatic vitamin K-dependent proteins in calcium homeostasis is implicated [100]. Calcium is a key building block of hydroxyapatite and important for calcification events to occur. Calcium supplementation might thus increase the risk of calcium-associated pathologies, such as ectopic calcification [100]. Proteins such as MGP act to prevent excess calcium from accumulating, thus limiting mineralization. Vitamin K deficiency reduces the activity of VKDPs. The hypothesis and data presented in this review further suggest that extrahepatic vitamin K deficiency cannot be alleviated with VK1 supplementation alone [101]. Although it remains a challenge to directly screen VK2, there is a strong argument that patients with osteoporosis, cardiovascular disease, and diabetes are deficient in VK2 [102,103,104]. The MK-7 isoform appears to have the greatest extrahepatic bioavailability [9]. It has been demonstrated that it has beneficial effects on both bone and cardiovascular health with a relatively low dose of 180 μg/day [105,106], with similar effects of much higher doses of VK1 [107,108]. These low doses can be additionally supported by VK2 intake from food. Although the mechanism remains unknown, it is plausible that the interaction of MK-7 with vitamin K-dependent proteins is stronger than that of other vitamin K isoforms. This may be due to the decreasing need for energy for longer menaquinone isoforms (MK-7, MK-8, MK-9) compared to VK1 and MK-4 (Figure 2) [108].

## 11. Conclusions and Next Steps

Differences between the pharmacokinetics of VK1 and VK2 in the human body are clear. The extrahepatic activity of VK2 has been demonstrated, although a detailed mechanistic understanding of VK2 activity is lacking from the literature. Having used the nine criteria set out for establishing bioactive RDI recommendation, VK2 clearly passes this. The bioactive VK2 is found relatively sporadically in a variety of fermented foods common to Western diets. Its specific concentration can vary drastically depending on factorial preparation methods, namely, which bacteria are used in fermentation processes. VK2 supplementation in various clinical trials have had either a significant improvement of health status or a strong correlation.

It is difficult to assess whether VK2 supplementation in itself will improve quality of life directly given the day-to-day variables by which individuals live their lives. This is a challenge faced by studies the world over, regardless of bioavailability and bioactivity. It is almost impossible to conclusively claim that a bioactive improves quality of life. Although these are limitations, it is known that VK2 supplementation, when used in a variety of clinical trials on bone and cardiovascular disease, results in a reduction in the development of disease. Therefore, it can be postulated that consistent consumption of VK2 can reduce the risk of occurrence of such aging diseases in the first place.

Establishing an RDI for VK2 could mean that food manufacturers have to use better quality bacteria in their fermentation processes to aid sales, as well as enable consumers to become more aware of the manufacturing processes in some of their favorite foods. There are no toxic consequences of VK2 overconsumption, so to supplement VK2 directly into other food sources would not cause any adverse effects and might be beneficial. Furthermore, the evidence clearly supports the benefit of high VK2 consumption. The modes by which VK2 levels can be assessed need to be standardized. KEQAS is spearheading this, and LC-MS/MS might be the answer.

In this review article, we have provided evidence based upon basic and clinical sciences for establishment of an RDI for VK2. The next steps would be for scientific and food policy makers to review the literature on the current state of VK2 research, given the nature of VK2 action on decreasing the development of diseases commonly linked to aging. Establishing an RDI for VK2 may have a significant impact in improving health the world over. This would reduce the socioeconomic consequences of an aging population by reducing the development of cardiovascular diseases, bone loss, and potentially, other age-related diseases.

## Figures and Tables

**Table 1:** Vitamin K1 and K2 content in various food sources.

Food Category	Food Source	Vitamin K1 Content per 100 g of Food Sample (µg)	Vitamin K2 Content per 100 g of Food Sample (µg)
Prepared vegetables	Natto (fermented soybeans)	32.1	108.9
	Roasted soybeans	57.3	Not compared in the study
	Sauerkraut	22.4	5.5
Vegetables	Collards	706	Not compared in the study
	Turnip	568	Not compared in the study
	Broccoli	146.7	Not compared in the study
	Spinach	96.7	Not compared in the study
	Kale	73.3	Not compared in the study
	Carrot	25.5	Not compared in the study
Fruits	Dried prunes	51.1–68.1	Not compared in the study
	Kiwifruit	33.9–50.3	Not compared in the study
	Avocado	15.7–27.0	Not compared in the study
	Blueberries	14.7–27.2	Not compared in the study
	Blackberries	14.7–25.1	Not compared in the study
	Grapes red and green	13.8–18.1	Not compared in the study
	Dried figs	11.4–20.0	Not compared in the study
Nuts	Pine nuts	33.4–73.7	Not compared in the study
	Cashews	19.4–64.3	Not compared in the study
	Pistachios	10.1–15.1	Not compared in the study
Cheese	Roquefort	6.56	38.1
	Pecorino	5.56	93.7
	Brie	4.92	12.5
	Boursin	4.55	11.1
	Norvegia	4.37	41.5
	Stilton	3.62	49.4
	Münster	2.1	80.1
	Camembert	2.5	68.1
	Gamalost	0.18	54.2
	Emmental	2.41	43.3
	Raclette	1.55	32.3
Meat	Beef liver	2.3	11.2
	Beef meat	0.02	1.89
	Minced meat	1.1	7.6
	Chicken meat	Not detected in the study	10.1
	Pork meat	Not detected in the study	1.4
	Pork liver	Not detected in the study	1.8
Fish	Mackerel	0.5	0.6
	Eel	1.3	63.1
	Plaice	Not detected in the study	5.3
	Prawns	Not detected in the study	0.19
	Salmon	0.13	0.6
